# Can Intradetrusor OnabotulinumtoxinA Injections Alter Heart Function in Patients with Cardiac Arrhythmia?

**DOI:** 10.3390/jcm7090263

**Published:** 2018-09-09

**Authors:** Pawel Miotla, Pawel Olejniczak, Konrad Futyma, Andrzej Wrobel, Michal Tomaszewski, Michal Bogusiewicz, Sara Wawrysiuk, Ewa Markut-Miotla, Tomasz Rechberger

**Affiliations:** 1 Second Department of Gynaecology, Medical University of Lublin, ul. Jaczewskiego 8, 20-954 Lublin, Poland; futymakonrad@mp.pl (K.F.); andrzejwrobel@umlub.pl (A.W.); michalbogusiewicz@umlub.pl (M.B.); sarawawrysiuk@umlub.pl (S.W.); tomaszrechberger@umlub.pl (T.R.); 2 Department of Urology, SPZOZ Staszow, 11 Listopada 78, Staszów 28-200, Poland; 3 Department of Cardiology, Medical University of Lublin, ul. Jaczewskiego 8, 20-954 Lublin, Poland; mdtomaszewski@wp.pl; 4 Department of Paediatric Pulmonology and Rheumatology, Medical University of Lublin, ul. Chodźki 1, 20-093 Lublin, Poland; ewamarkutmiotla@umlub.pl

**Keywords:** cardiac arrhythmia, overactive bladder, onabotulinumtoxinA injections, botulinum toxin A, urinary incontinence

## Abstract

The prevalence of overactive bladder (OAB) increases with age and can be associated with other co-morbidities, such as cardiac arrhythmia. Unfortunately, commonly used anticholinergic drugs for OAB can affect the cardiovascular system, leading to tachycardia. However, there are no data, which consider the influence of intradetrusor onabotulinumtoxinA injections on heart function in idiopathic OAB patients. The aim of the present study was to evaluate the influence of intradetrusor onabotulinumtoxinA injections on electrocardiogram (ECG) parameters. Additionally, changes in ECG were analyzed in OAB patients without cardiac arrhythmia. Thirty-one patients with cardiac arrhythmia and 31 participants without irregular heart rate (HR) completed the study. ECG measurements were performed in supine positions 2 h before onabotulinumtoxinA injections, 1 h after treatment, and at 2 weeks of follow-up. At week 6, a phone-call survey was conducted to collect data about adverse events. OnabotulinumtoxinA injections were performed with rigid cystoscopy under local anesthesia. We did not observe any clinically significant changes in the analyzed ECG parameters between consecutive measurements. While a slight increase of HR was observed in patients without cardiac arrhythmia, it remained within normal range. Intravesical onabotulinumtoxinA injections are, hence, safe for female patients with cardiac arrhythmia and do not significantly influence changes in ECG.

## 1. Introduction

Overactive bladder (OAB) has a complex set of symptoms, such as urgency, frequency, nocturia, and urge incontinence, which can significantly affect one’s quality of life [1]. The overall prevalence of OAB in women is reported at about 16.9%, and it incomparably increases with age [2]. Ageing also affects the cardiovascular system and can lead to heart disorders and arrhythmia, and supraventricular premature beats are observed in 5–10% of all individuals older than 60 years, while atrial fibrillation can affect 6% of those >65 years [3]. Therefore, prescribing pharmacotherapy for OAB treatment should include recognizing their potential influence on the cardiovascular system.

The main treatment method and first-line pharmacological options for OAB are antimuscarinic drugs and mirabegron. However, intradetrusor injections of onabotulinumtoxinA are approved as a second line of pharmacological treatment for OAB [4,5]. Antimuscarinic drugs and mirabegron are considered to be highly effective [6]. Still, due to the fact that muscarinic receptors are distributed throughout the whole body, antimuscarinic drugs present many side effects, including those concerning the heart [7]. The most common adverse events (AEs) are dry mouth, constipation, dry eyes, blurred vision, dizziness, and tachycardia [8].

Tolterodine, solifenancin, and oxybutynin are the antimuscarinic drugs most often used in the treatment of OAB. Each exhibits a slightly different spectrum of adverse events due to different muscarinic receptor selectivity. Tolterodine has a similar degree of selectivity for all muscarinic (M) receptors, whereas solifenacin has a greater level of selectivity for the M3 than for the M2 receptor [9]. Non-selective antimuscarinics (such as tolterodine) tend to increase the heart rate (HR) more than selectives [8]. Oxybutynin is considered to have the most significant spectrum of anticholinergic adverse effects (up to 80% of all patients), which is also connected with a high discontinuation rate [10].

Andersson et al. determined the rate of cardiovascular comorbidity in patients who were OAB-treated and untreated with antimuscarinic drugs, and no statistically significant difference was found. However, patients who were previously exposed to antimuscarinic drugs had an elevated heart rate prior to the experiment [11]. A further study by Balachandran et al. investigated 279 individuals with no previous cardiac arrhythmia. Herein, the estimated risk of developing palpitations was 2.9%, although the palpitations were considered to be short-lived and reversible in all cases [12]. Therefore, there is an unmet clinical need to identify therapeutics which are effective and well-tolerated by the older population.

OnabotulinumtoxinA prevents acetylcholine release at the neuromuscular junction, inducing muscle paralysis [13]. Although intradetrusor injections of onabotulinumtoxinA are considered to be highly effective in the treatment of OAB, the procedure is invasive and requires performing cystoscopy. The common potential adverse events after intravesical administration of the botulinum toxin A includes urinary tract infection, increased post-void residual volumes, and even urinary retention. At times, this last case requires clean intermittent self-catheterisation (CISC) [14]. With regard to cardiac function, botulinum toxin A is known to have a positive effect on temporary and long-term suppression of atrial fibrillation when injected into the epicardial fat pads [15,16,17].

Very little is known about systemic side effects, including cardiac function, following botulinum toxin A injections. Mehnert et al. observed changes in heart rate after an onabotulinumtoxinA intradetrusor injection to determine the systemic effect on cardiac function in patients with a neurogenic bladder but without co-existing heart diseases. The study showed no effect on the resting state function of the heart, though it had some limitations. One was the lack of patients with cardiac disorders who might be more sensitive towards developing some potential abnormalities in electrocardiograms after treatment [18]. Therefore, it would be attractive to investigate the influence of onabotulinumtoxinA injections on heart function in patients with pre-existing cardiac arrhythmias.

Hence, the aim of the study was to evaluate the influence of intravesical onabotulinumtoxinA on cardiac function in female OAB patients with cardiac arrhythmia. This was based on changes in heart rate and QTc interval (a measure of the time between the start of the Q wave and the end of the T wave in the heart`s electrical cycle, corrected for heart rate) on electrocardiograms, as it has previously been shown that elevated heart rate and prolonged QTc intervals have been identified as independent predictors of cardiovascular events [19,20,21].

## 2. Materials and Methods

**Figure 1:**
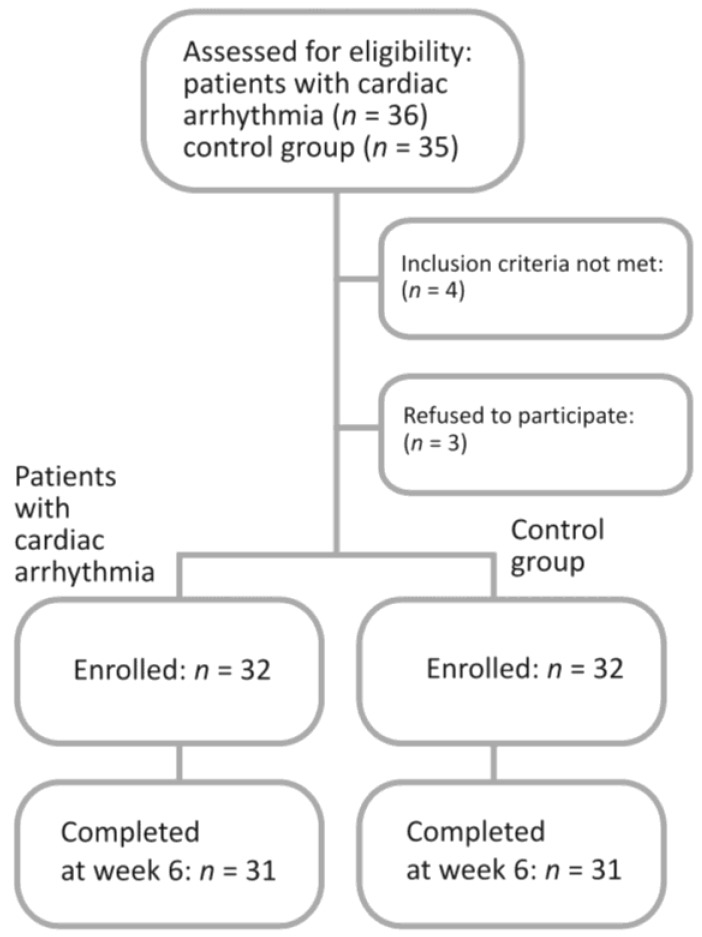
Flowchart of the participants in the study.

This was a prospective, case-controlled, single-center study. The study was conducted in accordance with the Declaration of Helsinki, and the protocol was approved by our local institutional ethical committee (No. KE-0254/74/2015). Before inclusion into the study, all patients, who were women of European descent, gave written informed consent. From June 2016 to October 2017, seventy-one patients with idiopathic OAB were approached to participate in this study. Patients were divided into two age-matched sub-groups—those with a diagnosis of cardiac arrhythmia and without cardiac arrhythmia. Patients with cardiac arrhythmia (extra beats, supraventricular tachycardia, ventricular arrhythmia) were on stable doses of antiarrhythmic drugs (beta-blockers, procainamid, amiodaron, or propafenon) for at least one month. The precise inclusion and exclusion criteria are summarized in Table 1. The final analysis included data collected from sixty-two OAB female patients—31 women with cardiac arrhythmia and 31 age-matched participants without cardiac arrhythmia who were enrolled into the control group to enable additional comparisons (Figure 1).

During the study, 12-lead electrocardiograms were performed according to the following schedule: 2 h before the onabotulinumtoxinA injection procedure, 1 h after treatment (or later if a patient reported the feeling of an unpleasant sensation in their bladder) and 2 weeks after the day of treatment. As the onset of the botulinum toxin A effect starts within the first 2 weeks after the neurotoxin injection, we decided to schedule the first follow-up visit at week 2 [23,24,25]. 

All ECGs were recorded in the supine position, with a paper speed of 25 mm/s, and in a rested and calm state for at least 15 min. Heart rate was determined as the number of beats per min (bpm) and was considered as accelerated when it was more than 100 bpm. The following parameters were measured: PR (a measure of the time between the beginning of the P wave and the beginning of the R wave), PQ (a measure the time between the beginning of the P wave and the beginning of the QRS complex), QT (a measure of the time between the start of the Q wave and the end of the T wave), QTc intervals and QRS (a measure of the time between the start of the Q wave and the end of the S wave), duration according to the rules described previously [26]. The QTc interval was calculated using the Bazett formula as follows: QTc = QT/√RR, where RR represents the R–R distance. All intervals and QRS complexes were manually assessed and measured in milliseconds (ms) in three cycles in each lead. Prolonged PQ and QTc intervals were determined as ≥200 ms and ≥460 ms, respectively. 

Normal values for intervals for ECG records were as follows: PR interval: 120–200 ms; PQ interval: 120–200 ms; QRS complex: 80–100 ms; QT interval: <420 ms; QTc interval: <460 ms [27,28,29].

Changes in the above-mentioned ECG parameters and ventricular rate were analyzed between each recording. Lack of sinus rhythm or relevant supraventricular arrhythmia (atrial tachycardia or atrial fibrillation), relevant ventricular arrhythmia (ventricular tachycardia or ventricular fibrillation, torsade de pointes), as well as atrioventricular block types II and III were considered as clinically unacceptable changes.

Additionally, blood pressure (BP) was assessed among participants at follow-up visits. Herein, it was measured after 15 min of rest in a sitting position on the non-dominant arm, according to the previously described schedule (2 h before the onabotulinumtoxinA injection procedure, 1 h after treatment (or later if a patient reported the feeling of an unpleasant sensation in their bladder) and 2 weeks after the day of treatment).

After 6 weeks, the patients were asked during a phone-call survey about any abnormalities. Patients were also advised to report any cardiac arrhythmia between all follow-up visits. 

At the day of the treatment, gynaecological examination was performed on all participants after ECG measurements as a routine procedure to exclude pelvic organ prolapse or other pathologies in their minor pelvis which could potentially influence bladder function. Moreover, at week 2, the patients were subjected to an additional urine dipstick assessment for screening of urinary tract infection, as well as a post-void residual measurement with abdominal, three-dimensional ultrasonography.

Screening for UTI was performed before the onabutolinumtoxinA injections were given. If the test results came back negative, the bladder was instilled with 100 mL solution of 2% lidocaine to provide local anaesthesia to the bladder wall. After 30 min, the bladder was emptied and rinsed with 0.9% sodium chloride (NaCl) solution. One hundred units of onabotulinumtoxinA (Botox, Allergan) was dissolved in 10 mL of 0.9% NaCl directly before the procedure and was administered during rigid cystoscopy in 20 intradetrusor injections in 2–3 horizontal lines, except the trigone. As was previously described, the tip of the needle (25-gauge injeTAK 198 DIS-Laborie) was inserted 3 mm into the bladder wall [30]. A single dose of fosfomycin tromethamine (3 g administered orally) was recommended as an antibiotic prophylaxis.

Two patients (one in each sub-group) were unable to attend the follow-up visit at week 2, and, therefore, were not included into the final analysis.

The primary outcome measures were heart rate and QT/QTc intervals. The secondary outcome parameters included changes in PR, PQ intervals, and QRS complex.

Statistical analysis was performed with Statistica Statsoft, package version 12, using the *χ^2^* test ANOVA with post-hoc tests and the Student *t* test, as appropriate. A *p* value of < 0.05 was defined as being statistically significant. Analysis of the power of the statistical tests used showed that in the case of comparison of the mean heart rate after the onabotulinumtoxinA injections between the study and control groups, the power exceeded 90% (two-sided, alpha = 0.05). 

## 3. Results

Baseline demographic characteristics were similar between groups and are summarized in Table 2.

Sixty-two age-matched patients completed the study, including the follow-up visit conducted by phone-call at week 6 (Figure 1). None of the patients in either study sub-group developed tachycardia or the prolonged QTc interval. In 4 patients (2 in each subgroup), prolonged PQ interval was observed—however, this alteration was present before intravesical onabotulinumtoxinA administration. We did not observe any worsening in ECG records and clinical status after the treatment when compared to the baseline visit. All patients had normal sinus rhythm in both study groups. In the arrhythmia group, we observed prior to the procedure: supraventricular extrasystolic beats (1 patient), ventricular extrasystolic beats (1 patient), left bundle branch block (1 patient), incomplete right bundle branch block (1 patient), and atrioventricular block type I (1 patient). We did not observe any worsening of these abnormalities during the study. Moreover, none of the patients who participated in the study developed new-onset arrhythmia.

The only statistically apparent finding was a slight increase in HR in the control group when comparing ECG performed 1 h after injections and at the follow-up visit in week 2. Still, these results remained within the normal range. We did not observe any significant differences in analyzed ECG intervals or QRS complex within each subgroup at subsequent measurements, as well as when the cardiac arrhythmia and control groups were compared. 

**Figure 2:**
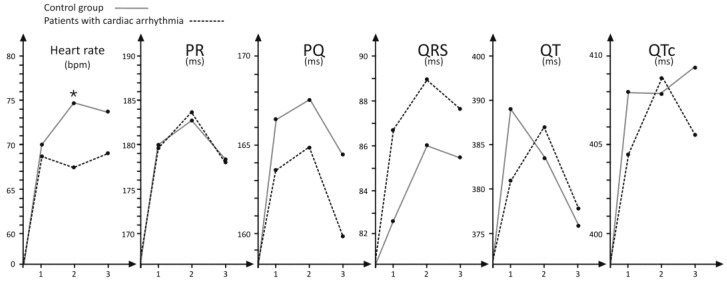
Comparison of ECG parameters between patients with cardiac arrhythmia and control groups during consecutive measurements. (1) 2 h pre-onabotulinumtoxinA injection procedure; (2) 1 h post-treatment (or later if an unpleasant sensation in their bladder was reported); (3) 2 weeks post-day-of-treatment; * *p* < 0.001; PR, a measure of the time between the beginning of the P wave and the beginning of the R wave; PQ, a measure the time between the beginning of the P wave and the beginning of the QRS complex; QRS, a measure of the time between the start of the Q wave and the end of the S wave; QT, a measure of the time between the start of the Q wave and the end of the T wave; QTc, a measure of the time between the start of the Q wave and the end of the T wave in the heart`s electrical cycle, corrected for heart rate.

Detailed data on the ECG findings is presented in Table 3 and Figure 2.

Furthermore, a slight increase in the mean heart rate (from 71 bpm to 74.7 bpm, *p* < 0.05) was observed in the control group when baseline and post-procedure ECG measurements were compared. However, this alteration is unlikely to be relevant, from a clinical point of view.

During the phone-call survey at week 6, none of the patients reported any subjective disturbances in heart rate or other cardiologic complaints. In addition, systolic and diastolic blood pressure did not differ significantly between the investigated groups at any point in the study. In both groups, BP was slightly lower at 30 min after the onabotulinumtoxinA injection, in comparison to the initial measurement. There were no significant differences in the mean BP measurements taken after the botulinum toxin A injection (Table 4).

### Other Safety Measurements

Urinary tract infection (UTI) was recognized by way of urine dipstick testing (positive nitrates, leukocytes, and erythrocytes) in only one patient, and she was from the control group. The patient was advised to have a urine culture performed, and antibiotic treatment was administered, according to the results, by a general practitioner.

At the follow-up visit in week 2, 5 patients (3 (9.6%) in the cardiac arrhythmia group and 2 (6.4%) in the control group, respectively) were found to have developed increased post-void residual volume. However, none of the patients required CISC. The values of residual urine did not exceed 200 mL.

## 4. Discussion

Intravesical onabotulinumtoxinA injection might be an attractive, alternative option for elderly OAB patients. This is because of the lack of many adverse events associated with oral antimuscarinics therapy [31]. In comparison with other pharmacotherapies for OAB, onabotulinumtoxinA (100 U) caused the greatest reduction in urgency, frequency, and urinary incontinence episodes. Even at 12 weeks post-injection, these improvements are significantly higher when compared to other pharmacological treatment options [32]. However, all patients should still be fully informed about the potential adverse events associated with onabotulinumtoxinA injections.

To the best of our knowledge, this study is the first to investigate the influence of onabotulinumtoxinA injections on heart function in OAB patients with cardiac arrhythmia. In the study, we assessed the ECG alterations in OAB women with cardiac arrhythmia (the study group) and without such abnormalities (the control group). 

As the prevalence of QTc prolongation in patients varies from 5% to 11% [33], based on previously published data which has confirmed that the prolonged QTc interval is a predictor of cardiovascular events [19,20,21], we decided to avoid administering any drug influencing the extension of the QTc interval. Due to the increasing rate of resistance to fluoroquinolones and their potential negative effect on ECG alterations, we introduced the prophylaxis of UTI with fosfomycine tromethamine [33,34]. Such a strategy enabled the exclusion of the potential negative influence of antibiotic prophylaxis on ECG, and could be more effective in the context of UTI prevention.

In our study, we did not observe any clinically significant changes in analyzed ECG measurements. The only finding was an increase in the mean heart rate observed between pre- and post-injection ECGs—however, HR was still within normal range. We can speculate that this issue could be incidental or that it is associated with certain psychological factors. A lack of the systemic influence of intradetrusor onabotulinumtoxinA injections on heart function is consistent with previously published research conducted in patients suffering from a neurogenic bladder [18].

We did observe higher values of systolic and diastolic blood pressure before the procedure when compared to subsequent follow-up assessments. That could be associated with pre-procedure emotional stress. In addition, we did not observe an increase of BP—however, such has been published in one case report in which hypertension appeared at 7 days post-intravesical injections of 300 units of onabotulinumtoxinA in a patient with a neurogenic bladder. Interestingly, this adverse event vanished after 4–5 months—a time period which is similar to the duration of the therapeutic effect of botulinum toxin A on bladder function observed in this present case [35].

We did not observe any other significant adverse consequences of onabotulinumtoxinA injections, except a transient increase in post-void residual volume. Herein, the incidence of this AE did not exceed previously published data [36]. 

The strengths of the study include the pragmatic setting, inclusion of participants with cardiac arrhythmia, as well as a good response rate at follow-up visits. Limitations include the single-center results, the relatively small number of patients, and lack of male patients in the study group. Another obvious limitation was the lack of patients’ selection according to various types of arrhythmia. This pivotal issue will definitely be addressed in future studies.

OAB can be treated in many ways, and it is important to keep in mind the adverse events which may be induced by the most common treatment options, such as the administration of antimuscarinic drugs, mirabegron, or intraderusor botulinum toxin A. In our study, we did not observe any unwanted cardiac events after onabotulinumtoxinA injections—either in the study group or in the control group. We also did not observe any increase in heart rate and QTc interval, or any other pathological changes in ECG in the patients with cardiac arrhythmia. Nonetheless, it is worth remembering that the history of cardiac dysfunction may have an influence on the course of treatment and on the occurrence of cardiac side effects. Moreover, patients with an existing cardiac disease are more prone to developing changes in ECG or cardiac events during OAB treatment. Due to the risk of significant side effects, the treatment of OAB should be carefully considered together with a patient’s co-morbidities.

## 5. Conclusions 

This observational study shows that intradetrusor onabotulinumtoxinA injections (100 U) seem to be safe for female patients in terms of cardiac arrhythmia. Such treatment does not trigger any significant changes in heart rate or electrocardiographic abnormalities, or subjective feelings of irregular heart rate.

## Figures and Tables

**Table 1:** Inclusion and exclusion criteria of the study for overactive bladder (OAB) patients.

Inclusion Criteria	Exclusion Criteria
Non-pregnant women ≥18 years of age	Urinary tract infection and/or haematuria (ascertained via urine dipstick)
Idiopathic OAB symptoms for at least 6 months	Bladder or pelvic tumours
	Bladder or kidney stones
≥8 micturitions/24 h	Neurologic disorders affecting bladder function
≥1 urgency urinary incontinence/24h	Bladder painful syndrome
	Unexplained pelvic pain
	Post-void residual volume over 100 mL
	Measured through ultrasonography
Lack of efficacy (duration of treatment ≥1 month) or intolerance of antimuscarinics and/or mirabegron	Allergy to lidocaine
	Uncontrolled systemic diseases, i.e., diabetes, hyperthyreosis
	Previous onabotulinumtoxinA treatment
Stage 0 or 1 in pelvic organ prolapse quantification (POP-Q) scale [22]	Stage ≥2 in POPQ scale [22]
	Electrolyte abnormalities
	Cardiac pacemaker

**Table 2:** Demographic characteristics of overactive bladder (OAB) patient groups.

Variable	Patients with Cardiac Arrhythmia (*n* = 31)	Patients without Cardiac Arrhythmia (*n* = 31)	*p*
Age (years)	58.9 ± 13.4	58.7 ± 13.0	NS
BMI (kg/m^2^)	27.8 ± 4.1	27.9 ± 3.7	NS
Parity	1.7 ± 0.8	1.9 ± 0.8	NS
Menopause	24 (77.4)	25 (80.6)	NS

Continuous variables are presented as the mean ± SD, categorical variables are presented as number and %; NS, non-significant; BMI, body mass index.

**Table 3:** Results of electrocardiogram (ECG) parameters in overactive bladder (OAB) patients during the study.

Variable		Patients with Cardiac Arrhythmia (*n* = 31)	Patients without Cardiac Arrhythmia (*n* = 31)	*p*
Heart rate (bpm)	(1)	68.8 ± 7.8	71.0 ± 6.2	NS
	(2)	67.5 ± 6.4	74.7 ± 7.0	<0.001
	(3)	69.0 ± 11.2	73.8 ± 6.3	<0.05
PR interval (ms)	(1)	179.5 ± 24.8	179.8 ± 17.8	NS
	(2)	183.7 ± 27.9	182.8 ± 21.5	NS
	(3)	178.0 ± 22.3	178.2 ± 16.7	NS
PQ interval (ms)	(1)	163.5 ± 21.9	166.4 ± 20.8	NS
	(2)	164.8 ± 28.4	167.5 ± 29.4	NS
	(3)	159.8 ± 21.7	164.3 ± 16.1	NS
QRS complex (ms)	(1)	88.3 ± 14.4	82.7 ± 7.9	NS
	(2)	89.0 ± 15.6	86.0 ± 10.3	NS
	(3)	87.7 ± 12.8	85.4 ± 9.2	NS
QT interval (ms)	(1)	381.1 ± 22.4	389.0±38.8	NS
	(2)	387.4 ± 25.2	383.5 ± 24.7	NS
	(3)	377.9 ± 28.6	375.8 ± 26.5	NS
QTc interval (ms)	(1)	404.3 ± 24.8	407.0 ± 23.8	NS
	(2)	408.8 ± 21.3	407.9 ± 18.7	NS
	(3)	405.6 ± 23.8	409.4 ± 19.6	NS

Continuous variables are presented as the mean ± SD; (1) 2 h pre-onabotulinumtoxinA injection procedure; (2) 1 h post-treatment (or later if an unpleasant sensation in their bladder was reported); (3) 2 weeks post-day-of-treatment; NS, non-significant; bpm, beats per min; ms, milliseconds; PR, a measure of the time between the beginning of the P wave and the beginning of the R wave; PQ, a measure the time between the beginning of the P wave and the beginning of the QRS complex; QRS, a measure of the time between the start of the Q wave and the end of the S wave; QT, a measure of the time between the start of the Q wave and the end of the T wave; QTc, a measure of the time between the start of the Q wave and the end of the T wave in the heart`s electrical cycle, corrected for heart rate.

**Table 4:** Results of blood pressure measurements in overactive bladder (OAB) patients during the study.

Variable		Patients with Cardiac Arrhythmia (*n* = 31)	*p*	Patients without Cardiac Arrhythmia (*n* = 31)	*p*
Systolic blood pressure (mmHg)	(1)	136.4 ± 13.3	1. vs. 2. <0.05 1. vs. 3. NS 2. vs. 3. NS	134.2 ± 13.1	1. vs. 2. <0.05 1. vs. 3. NS 2. vs. 3. NS
	(2)	133.2 ± 11.2		131.0 ± 11.9	
	(3)	133.2 ± 9.4		130.2 ± 8.3	
Diastolic blood pressure (mmHg)	(1)	85.3 ± 8.4	1. vs. 2. <0.05 1. vs. 3. NS 2. vs. 3. NS	84.0 ± 8.3	1. vs. 2. <0.05 1. vs. 3. NS 2. vs. 3. NS
	(2)	81.6 ± 6.1		81.9 ± 7.7	
	(3)	81.5 ± 6.1		79.2 ± 5.8	

Continuous variables are presented as the mean ± standard deviation. mmHg, millimeters of mercury; NS, non-significant; Legend: (1) 2 h pre-onabotulinumtoxinA injection procedure; (2) 1 h post-treatment (or later if an unpleasant sensation in their bladder was reported); (3) 2 weeks post-day-of-treatment.
